# Supplementary material for: Multifaced Role of Dual Herbal Principles Loaded-Lipid Nanocarriers in Providing High Therapeutic Efficacity
Source: Pharmaceutics. 2021 Sep 18;13(9):1511. doi: 10.3390/pharmaceutics13091511 (PMC8465057; doi:10.3390/pharmaceutics13091511)
Supplement: Supplementary file 1 [file pharmaceutics-13-01511-s001.zip › pharmaceutics-1369014-supplementary.pdf]

pharmaceutics

Supplementary Materials: Multifaced Role of Dual Herbal Principles Loaded-Lipid Nanocarriers in Providing High Therapeutic Efficacity

Ioana Lacatusu, Teodora Alexandra Iordache, Mirela Mihaila, Dan Eduard Mihaiescu, Anca Lucia Pop and Nicoleta Badea

Experimental method. High Resolution Mass Spectrometer with 15T superconducting magnet (solariX-XR, QqqFT-ICR HR, Bruker Daltonics) of Fourier-Transform-Ion-Cyclotron-Resonance (FT-ICR) type was used. The herbal extract was introduced by direct infusion, negative ESI ionization, with a sample flow rate of 120 μL/h, with a spray gas pressure (N_2) of 2.5 bar at 200 °C and a flow rate of 7 L/min. The spectra were recorded over a mass range between 122 and 1000 uam at a source voltage of 5500 V.

By ESI-HR-MS analysis of *Glycyrrhiza glabra* extract, the ion corresponding to *glycyrrhizic acid* in negative ionization was obtained at [M-1] 821.67, *m/z* (Figure S1). The parent ion of Glycyrrhizic Acid (protonated precursor ions $[M–H]^-$ from *m/z* 821.67 was also reported in several literature references [1,2].

A fragmentation of the precursor ion was also made, the fragmentation spectrum is shown in Figure S2.

The MS/MS spectrum was recorded using the fragmentation technique (FSCID source) with a 30 V collision energy using the precursor ion *m/z* = 821.68 and a narrow isolation window of 0.03 *m/z*; the RF collision amplitude of 1700 Vpp and the RF frequency was 1.4 MHz.

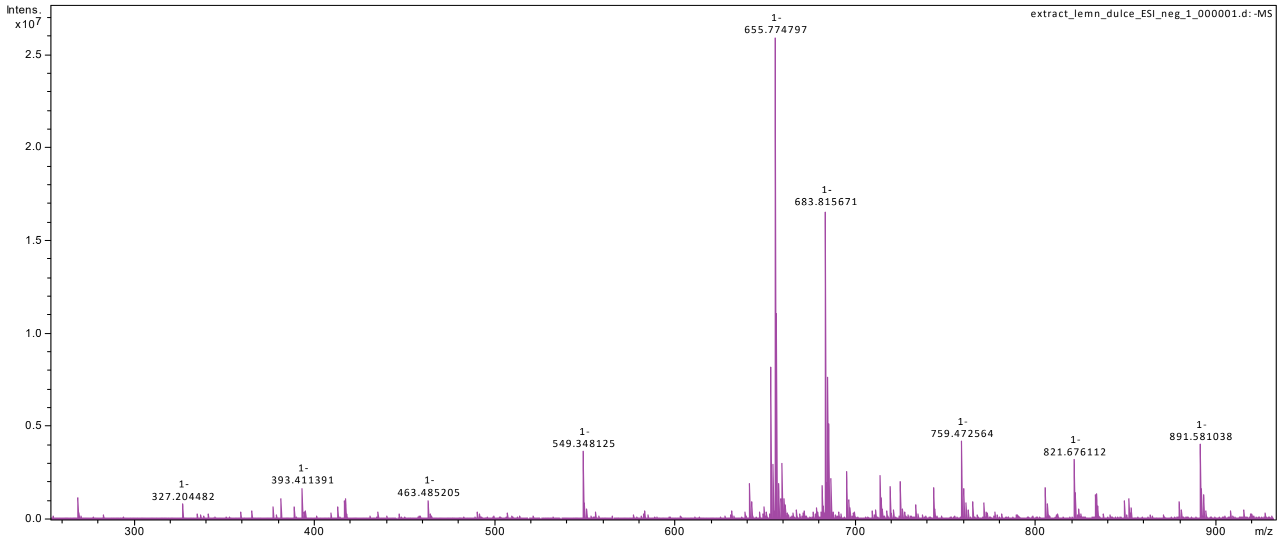

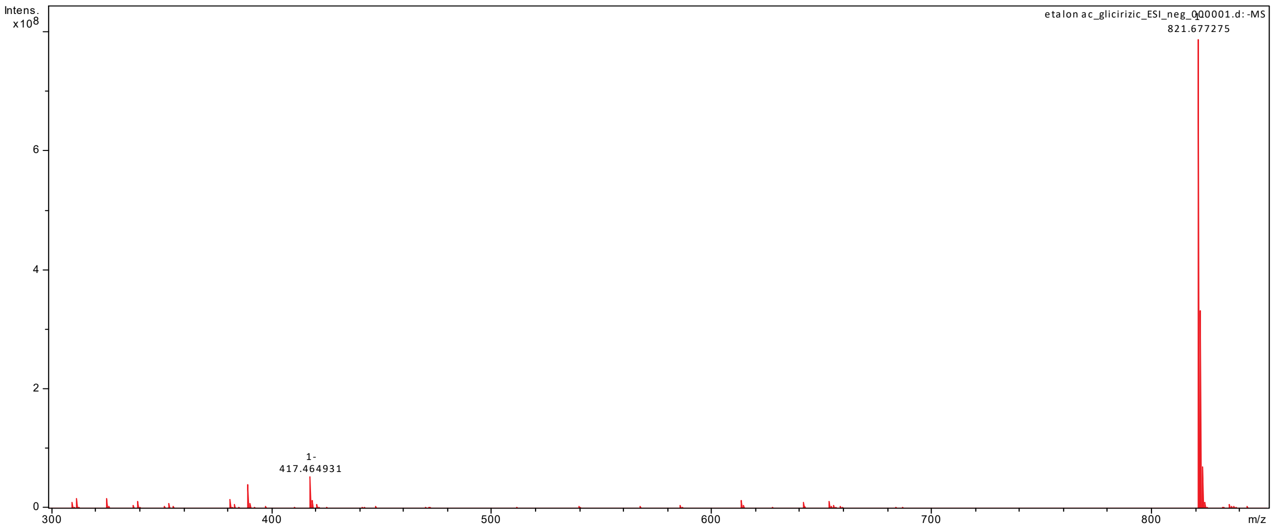

Figure S1. ESI-HR-Mass Spectrum of: (**a**). *Glycyrrhiza glabra extract* (GlyG) with Glycyrrhizic Acid parent ion (protonated precursor ions [M–H]- at m/z 821.67; (**b**). Glycyrrhizic Acid (standard), with protonated precursor ions [M–H]⁻ at *m*/*z* 821.67.

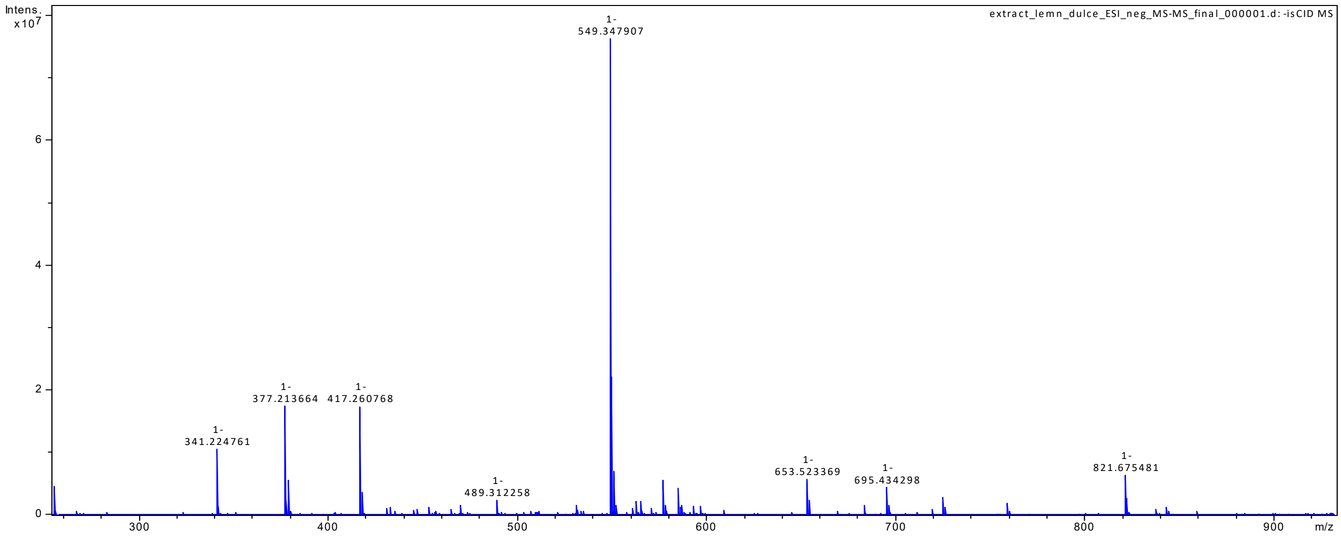

Figure S2. MS/MS Spectrum of *Glycyrrhiza glabra extract* obtained by fragmentation technique, using the precursor ion *m*/*z* = 821.68.

References

1. Ahmad, N.; Al-Subaiec, A.M.; Ahmad, R.; Sharma, S.; Alam, M.A.; Ashafaq, M; Rub, R.A.; Ahmad, F.J. Brain-targeted glycyrrhizic-acid-loaded surface decorated nanoparticles for treatment of cerebral ischaemia and its toxicity assessment. *Nanomed. Biotechnol.* **2019**, *47*, 475–490. doi: 10.1080/21691401.2018.1561458.
2. Qiao, X.; Song, W.; Ji S., Wang; Q., Guo, D.; Ye, M. Separation and characterization of phenolic compounds andtriterpenoid saponins in licorice (Glycyrrhiza uralensis) using mobilephase-dependent reversed-phase reversed-phase comprehensivetwo-dimensional liquid chromatography coupled with massspectrometry. *J. Chromatogr. A.* **2015**, *1402*, 36–45.
